# 2-(Thio­phen-2-yl)ethyl 4-methyl­benzene­sulfonate

**DOI:** 10.1107/S1600536811023907

**Published:** 2011-06-25

**Authors:** Yan-Shu Liang, Bing-Ni Liu, Mo Liu, Deng-Ke Liu

**Affiliations:** a Tianjin University of Commerce, Tianjin 300134, People’s Republic of China; b Tianjin Institute of Pharmaceutical Research, Tianjin 300193, People’s Republic of China

## Abstract

In the title mol­ecule, C_13_H_14_O_3_S_2_, the thio­phene and benzene rings form a dihedral angle of 13.86 (13)°. In the crystal, weak inter­molecular C—H⋯O hydrogen bonds link the mol­ecules into layers parallel to the *ab* plane.

## Related literature

The title compound is an inter­mediate in the synthesis of the anti­platelet agent clopidogrel (systematic name (+)-(*S*)-methyl 2-(2-chloro­phen­yl)-2-(6,7-dihydro­thieno[3,2-*c*]pyridin-5(4*H*)-yl)acetate). For background to the bioactivity and applications of clopidogrel, see: Raju *et al.* (2008 ▶). For the synthesis of the title compound, see: Sajja *et al.* (2007 ▶).
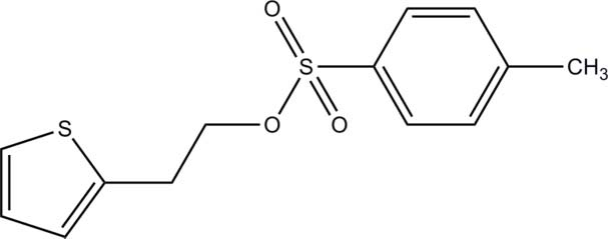


## Experimental

### 

#### Crystal data

C_13_H_14_O_3_S_2_
*M* *_r_* = 282.36
Monoclinic, 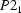

*a* = 8.6130 (9) Å
*b* = 5.9961 (4) Å
*c* = 13.1284 (12) Å
β = 97.935 (19)°
*V* = 671.52 (10) Å^3^
*Z* = 2
Mo *K*α radiation
μ = 0.39 mm^−1^
*T* = 113 K
0.20 × 0.18 × 0.10 mm

#### Data collection

Rigaku Saturn CCD area-detector diffractometer
Absorption correction: multi-scan (*CrystalClear*; Rigaku/MSC, 2005 ▶) *T*_min_ = 0.926, *T*_max_ = 0.962
6939 measured reflections
3167 independent reflections
2365 reflections with *I* > 2σ(*I*)
*R*_int_ = 0.048

#### Refinement

*R*[*F* ^2^ > 2σ(*F* ^2^)] = 0.035
*wR*(*F* ^2^) = 0.088
*S* = 0.83
3167 reflections
164 parameters
1 restraint
H-atom parameters constrained
Δρ_max_ = 0.39 e Å^−3^
Δρ_min_ = −0.38 e Å^−3^
Absolute structure: Flack (1983 ▶), 1405 Friedel pairs
Flack parameter: 0.00 (8)

### 

Data collection: *CrystalClear* (Rigaku/MSC, 2005 ▶); cell refinement: *CrystalClear*; data reduction: *CrystalClear*; program(s) used to solve structure: *SHELXS97* (Sheldrick, 2008 ▶); program(s) used to refine structure: *SHELXL97* (Sheldrick, 2008 ▶); molecular graphics: *SHELXTL* (Sheldrick, 2008 ▶); software used to prepare material for publication: *CrystalStructure* (Rigaku/MSC, 2005 ▶).

## Supplementary Material

Crystal structure: contains datablock(s) global, I. DOI: 10.1107/S1600536811023907/cv5113sup1.cif

Structure factors: contains datablock(s) I. DOI: 10.1107/S1600536811023907/cv5113Isup2.hkl

Supplementary material file. DOI: 10.1107/S1600536811023907/cv5113Isup3.cdx

Supplementary material file. DOI: 10.1107/S1600536811023907/cv5113Isup4.cml

Additional supplementary materials:  crystallographic information; 3D view; checkCIF report

## Figures and Tables

**Table 1:** Hydrogen-bond geometry (Å, °)

*D*—H⋯*A*	*D*—H	H⋯*A*	*D*⋯*A*	*D*—H⋯*A*
C2—H2⋯O2^i^	0.95	2.58	3.523 (3)	174
C6—H6*B*⋯O2^ii^	0.99	2.41	3.161 (3)	132
C13—H13*A*⋯O3^iii^	0.98	2.58	3.496 (3)	155

Symmetry codes: (i) 
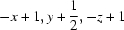
; (ii) 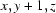; (iii) 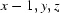.

**Fig. 1.**
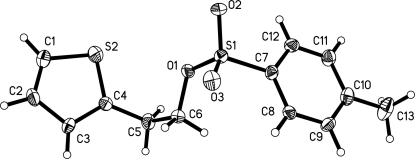
The molecular structure of (I), with the atom-numbering scheme and 50% probability displacement ellipsoids.

## supplementary crystallographic information

### Comment

Clopidogrel, a thienopyridine class inhibitor of P2Y12 ADP platelet receptor, has been found to be particularly useful in the treatment of coronary artery disease, peripheral vascular disease and cerebrovascular disease (Raju *et al.*, 2008). Herewith we present the crystal structure of the title compound (I) used as an intermediate in the synthesis of clopidogrel (Sajja *et al.*, 2007).

In (I) (Fig. 1), the dihedral angle formed between the benzene ring plane (r.m.s. deviation 0.0029 Å) and the thiophene ring plane (r.m.s. deviation 0.0025 Å) is 13.86 (13)°. The packing of the crystal is consolidated by the weak C—H···O interactions (Table 1).

### Experimental

5 g of 4-Methylbenzene-1-sulfonyl chloride and 30 ml of toluene were charged into a clean and dry reactor followed by cooling to about 5 °C. 3.4 g 2-(Thiophen-2-yl)ethanol was added at about 5 °C over about 20 minutes, followed by addition of 4.5 g of triethylamine over about 6 h. The reaction mixture temperature was raised to about 30 °C, followed by stirring for about 9 h. The reaction mass was filtered through a Nutsche filter and washed with 50 ml of toluene, and then, the reaction filtrate was transferred into another reactor followed by washing with 80 ml of water. Organic and aqueous layers were separated and the organic layer was distilled completely at about below 70 °C to afford 6.3 g of off-white solid as crude product. The solid was dissolved in methanol 30 ml at 20 °C, then colourless crystals were generated slowly.

### Refinement

All H atoms were positioned geometrically (C—H 0.95 - 0.99 Å) and refined using a riding model, with *U*_iso_(H) = 1.2 or 1.5*U*_eq_(C).

### Crystal data

**Table:**

C_13_H_14_O_3_S_2_	*F*(000) = 296
*M**_r_* = 282.36	*D*_x_ = 1.396 Mg m^−^^3^
Monoclinic, *P*2_1_	Mo *K*α radiation, λ = 0.71073 Å
Hall symbol: P 2yb	Cell parameters from 2573 reflections
*a* = 8.6130 (9) Å	θ = 2.4–27.9°
*b* = 5.9961 (4) Å	µ = 0.39 mm^−^^1^
*c* = 13.1284 (12) Å	*T* = 113 K
β = 97.935 (19)°	Prism, colourless
*V* = 671.52 (10) Å^3^	0.20 × 0.18 × 0.10 mm
*Z* = 2	

### Data collection

**Table:**

Rigaku Saturn CCD area-detector diffractometer	3167 independent reflections
Radiation source: rotating anode	2365 reflections with *I* > 2σ(*I*)
multilayer	*R*_int_ = 0.048
Detector resolution: 14.63 pixels mm^-1^	θ_max_ = 27.9°, θ_min_ = 2.4°
ω and φ scans	*h* = −11→11
Absorption correction: multi-scan (*CrystalClear*; Rigaku/MSC, 2005)	*k* = −7→7
*T*_min_ = 0.926, *T*_max_ = 0.962	*l* = −17→13
6939 measured reflections	

### Refinement

**Table:**

Refinement on *F*^2^	Secondary atom site location: difference Fourier map
Least-squares matrix: full	Hydrogen site location: inferred from neighbouring sites
*R*[*F*^2^ > 2σ(*F*^2^)] = 0.035	H-atom parameters constrained
*wR*(*F*^2^) = 0.088	*w* = 1/[σ^2^(*F*_o_^2^) + (0.0455*P*)^2^] where *P* = (*F*_o_^2^ + 2*F*_c_^2^)/3
*S* = 0.83	(Δ/σ)_max_ = 0.021
3167 reflections	Δρ_max_ = 0.39 e Å^−^^3^
164 parameters	Δρ_min_ = −0.38 e Å^−^^3^
1 restraint	Absolute structure: Flack (1983), 1405 Friedel pairs
Primary atom site location: structure-invariant direct methods	Flack parameter: 0.00 (8)

### Special details

**Table:**

Geometry. All e.s.d.'s (except the e.s.d. in the dihedral angle between two l.s. planes) are estimated using the full covariance matrix. The cell e.s.d.'s are taken into account individually in the estimation of e.s.d.'s in distances, angles and torsion angles; correlations between e.s.d.'s in cell parameters are only used when they are defined by crystal symmetry. An approximate (isotropic) treatment of cell e.s.d.'s is used for estimating e.s.d.'s involving l.s. planes.
Refinement. Refinement of *F*^2^ against ALL reflections. The weighted *R*-factor *wR* and goodness of fit *S* are based on *F*^2^, conventional *R*-factors *R* are based on *F*, with *F* set to zero for negative *F*^2^. The threshold expression of *F*^2^ > σ(*F*^2^) is used only for calculating *R*-factors(gt) *etc*. and is not relevant to the choice of reflections for refinement. *R*-factors based on *F*^2^ are statistically about twice as large as those based on *F*, and *R*- factors based on ALL data will be even larger.

### Fractional atomic coordinates and isotropic or equivalent isotropic displacement parameters (Å^2^)

**Table:**

	*x*	*y*	*z*	*U*_iso_*/*U*_eq_	
S1	0.19325 (6)	0.31172 (10)	0.75706 (4)	0.02115 (14)	
S2	0.22445 (7)	0.29779 (13)	0.39671 (4)	0.03087 (17)	
O1	0.15911 (18)	0.3941 (3)	0.64203 (11)	0.0213 (4)	
O2	0.1803 (2)	0.0753 (3)	0.75046 (13)	0.0330 (5)	
O3	0.33583 (19)	0.4105 (3)	0.80406 (12)	0.0278 (4)	
C1	0.3827 (2)	0.3018 (6)	0.33242 (16)	0.0278 (5)	
H1	0.4116	0.1822	0.2915	0.033*	
C2	0.4623 (3)	0.4953 (5)	0.34661 (18)	0.0275 (6)	
H2	0.5542	0.5244	0.3163	0.033*	
C3	0.3976 (2)	0.6547 (4)	0.41104 (16)	0.0193 (5)	
H3	0.4385	0.7991	0.4282	0.023*	
C4	0.2621 (3)	0.5599 (4)	0.44481 (16)	0.0201 (5)	
C5	0.1556 (3)	0.6739 (4)	0.51055 (16)	0.0263 (6)	
H5A	0.1611	0.8368	0.4993	0.032*	
H5B	0.0465	0.6263	0.4870	0.032*	
C6	0.1919 (3)	0.6291 (4)	0.62353 (17)	0.0221 (5)	
H6A	0.3035	0.6624	0.6477	0.027*	
H6B	0.1265	0.7255	0.6617	0.027*	
C7	0.0366 (3)	0.4163 (4)	0.81555 (16)	0.0202 (5)	
C8	0.0527 (3)	0.6179 (4)	0.86654 (16)	0.0207 (5)	
H8	0.1479	0.6996	0.8707	0.025*	
C9	−0.0732 (3)	0.7003 (4)	0.91203 (16)	0.0232 (5)	
H9	−0.0638	0.8400	0.9465	0.028*	
C10	−0.2126 (3)	0.5795 (5)	0.90745 (16)	0.0269 (6)	
C11	−0.2247 (3)	0.3748 (4)	0.85539 (17)	0.0261 (6)	
H11	−0.3190	0.2912	0.8518	0.031*	
C12	−0.1017 (2)	0.2926 (5)	0.80928 (15)	0.0233 (5)	
H12	−0.1110	0.1540	0.7739	0.028*	
C13	−0.3486 (3)	0.6686 (5)	0.9550 (2)	0.0415 (8)	
H13A	−0.4457	0.6455	0.9078	0.062*	
H13B	−0.3336	0.8284	0.9687	0.062*	
H13C	−0.3554	0.5901	1.0197	0.062*	

### Atomic displacement parameters (Å^2^)

**Table:**

	*U*^11^	*U*^22^	*U*^33^	*U*^12^	*U*^13^	*U*^23^
S1	0.0254 (3)	0.0184 (3)	0.0206 (3)	0.0022 (3)	0.0066 (2)	0.0007 (3)
S2	0.0249 (3)	0.0325 (4)	0.0362 (3)	−0.0035 (4)	0.0080 (2)	−0.0007 (4)
O1	0.0264 (9)	0.0206 (9)	0.0176 (8)	−0.0047 (7)	0.0059 (7)	−0.0013 (6)
O2	0.0464 (11)	0.0142 (11)	0.0429 (12)	0.0044 (8)	0.0224 (9)	0.0026 (8)
O3	0.0240 (9)	0.0355 (11)	0.0228 (9)	0.0015 (8)	−0.0004 (7)	0.0032 (8)
C1	0.0266 (12)	0.0343 (14)	0.0230 (11)	0.0024 (15)	0.0053 (9)	−0.0027 (15)
C2	0.0210 (13)	0.0330 (16)	0.0301 (13)	0.0011 (11)	0.0097 (10)	0.0086 (12)
C3	0.0179 (11)	0.0230 (15)	0.0163 (11)	0.0085 (10)	0.0000 (8)	0.0010 (10)
C4	0.0235 (12)	0.0206 (14)	0.0160 (11)	0.0033 (10)	0.0018 (9)	0.0051 (10)
C5	0.0282 (13)	0.0274 (16)	0.0245 (12)	0.0073 (11)	0.0080 (10)	0.0070 (11)
C6	0.0246 (12)	0.0189 (14)	0.0236 (12)	−0.0014 (10)	0.0056 (9)	−0.0005 (10)
C7	0.0245 (13)	0.0214 (14)	0.0149 (11)	0.0018 (10)	0.0032 (9)	0.0009 (10)
C8	0.0277 (13)	0.0196 (14)	0.0149 (11)	−0.0032 (10)	0.0041 (9)	0.0017 (9)
C9	0.0317 (13)	0.0192 (13)	0.0185 (11)	0.0031 (11)	0.0026 (9)	−0.0029 (11)
C10	0.0283 (14)	0.0343 (17)	0.0182 (12)	0.0025 (12)	0.0036 (10)	−0.0009 (11)
C11	0.0251 (13)	0.0309 (17)	0.0225 (12)	−0.0049 (10)	0.0039 (10)	−0.0049 (10)
C12	0.0300 (12)	0.0200 (13)	0.0196 (10)	−0.0027 (13)	0.0023 (8)	−0.0010 (12)
C13	0.0290 (14)	0.059 (2)	0.0373 (15)	0.0042 (15)	0.0080 (11)	−0.0180 (16)

### Geometric parameters (Å, °)

**Table:**

S1—O2	1.4236 (19)	C6—H6A	0.9900
S1—O3	1.4250 (17)	C6—H6B	0.9900
S1—O1	1.5776 (15)	C7—C8	1.379 (3)
S1—C7	1.758 (2)	C7—C12	1.396 (3)
S2—C1	1.700 (2)	C8—C9	1.399 (3)
S2—C4	1.708 (3)	C8—H8	0.9500
O1—C6	1.464 (3)	C9—C10	1.396 (3)
C1—C2	1.347 (4)	C9—H9	0.9500
C1—H1	0.9500	C10—C11	1.402 (4)
C2—C3	1.438 (3)	C10—C13	1.500 (3)
C2—H2	0.9500	C11—C12	1.382 (3)
C3—C4	1.423 (3)	C11—H11	0.9500
C3—H3	0.9500	C12—H12	0.9500
C4—C5	1.507 (3)	C13—H13A	0.9800
C5—C6	1.497 (3)	C13—H13B	0.9800
C5—H5A	0.9900	C13—H13C	0.9800
C5—H5B	0.9900		
			
O2—S1—O3	119.74 (12)	C5—C6—H6A	110.0
O2—S1—O1	104.53 (10)	O1—C6—H6B	110.0
O3—S1—O1	108.65 (9)	C5—C6—H6B	110.0
O2—S1—C7	108.87 (12)	H6A—C6—H6B	108.4
O3—S1—C7	109.28 (11)	C8—C7—C12	121.5 (2)
O1—S1—C7	104.69 (10)	C8—C7—S1	119.52 (18)
C1—S2—C4	92.64 (13)	C12—C7—S1	119.0 (2)
C6—O1—S1	116.46 (14)	C7—C8—C9	119.0 (2)
C2—C1—S2	111.8 (2)	C7—C8—H8	120.5
C2—C1—H1	124.1	C9—C8—H8	120.5
S2—C1—H1	124.1	C10—C9—C8	120.8 (2)
C1—C2—C3	115.0 (2)	C10—C9—H9	119.6
C1—C2—H2	122.5	C8—C9—H9	119.6
C3—C2—H2	122.5	C9—C10—C11	118.7 (2)
C4—C3—C2	108.6 (2)	C9—C10—C13	120.9 (3)
C4—C3—H3	125.7	C11—C10—C13	120.3 (2)
C2—C3—H3	125.7	C12—C11—C10	121.1 (2)
C3—C4—C5	126.0 (2)	C12—C11—H11	119.5
C3—C4—S2	111.98 (17)	C10—C11—H11	119.5
C5—C4—S2	121.96 (18)	C11—C12—C7	118.9 (3)
C6—C5—C4	115.19 (19)	C11—C12—H12	120.5
C6—C5—H5A	108.5	C7—C12—H12	120.5
C4—C5—H5A	108.5	C10—C13—H13A	109.5
C6—C5—H5B	108.5	C10—C13—H13B	109.5
C4—C5—H5B	108.5	H13A—C13—H13B	109.5
H5A—C5—H5B	107.5	C10—C13—H13C	109.5
O1—C6—C5	108.57 (19)	H13A—C13—H13C	109.5
O1—C6—H6A	110.0	H13B—C13—H13C	109.5
			
O2—S1—O1—C6	−169.41 (15)	O3—S1—C7—C8	22.3 (2)
O3—S1—O1—C6	−40.48 (18)	O1—S1—C7—C8	−93.96 (19)
C7—S1—O1—C6	76.18 (17)	O2—S1—C7—C12	−25.2 (2)
C4—S2—C1—C2	0.0 (2)	O3—S1—C7—C12	−157.67 (17)
S2—C1—C2—C3	−0.4 (3)	O1—S1—C7—C12	86.1 (2)
C1—C2—C3—C4	0.7 (3)	C12—C7—C8—C9	−0.6 (3)
C2—C3—C4—C5	−177.6 (2)	S1—C7—C8—C9	179.48 (17)
C2—C3—C4—S2	−0.6 (2)	C7—C8—C9—C10	0.9 (3)
C1—S2—C4—C3	0.37 (17)	C8—C9—C10—C11	−0.6 (3)
C1—S2—C4—C5	177.50 (18)	C8—C9—C10—C13	−179.2 (2)
C3—C4—C5—C6	−94.9 (3)	C9—C10—C11—C12	0.0 (3)
S2—C4—C5—C6	88.4 (2)	C13—C10—C11—C12	178.6 (2)
S1—O1—C6—C5	178.83 (14)	C10—C11—C12—C7	0.3 (3)
C4—C5—C6—O1	−67.3 (3)	C8—C7—C12—C11	0.0 (3)
O2—S1—C7—C8	154.71 (18)	S1—C7—C12—C11	179.91 (17)

### Hydrogen-bond geometry (Å, °)

**Table:**

*D*—H···*A*	*D*—H	H···*A*	*D*···*A*	*D*—H···*A*
C2—H2···O2^i^	0.95	2.58	3.523 (3)	174
C6—H6B···O2^ii^	0.99	2.41	3.161 (3)	132
C13—H13A···O3^iii^	0.98	2.58	3.496 (3)	155

Symmetry codes: (i) −*x*+1, *y*+1/2, −*z*+1; (ii) *x*, *y*+1, *z*; (iii) *x*−1, *y*, *z*.

## References

[bb1] Flack, H. D. (1983). *Acta Cryst.* A**39**, 876–881.

[bb2] Raju, N. C., Eikelboom, J. W. & Hirsh, J. (2008). *Nat. Clin. Pract. Cardiovasc. Med.* **5**, 766–780. 10.1038/ncpcardio1372 18957959

[bb3] Rigaku/MSC (2005). *CrystalClear* and *CrystalStructure* Rigaku/MSC Inc., The Woodlands, Texas, USA.

[bb4] Sajja, E. S., Anumula, R. R., Gilla, G. & Madivada, L. R. (2007). US Patent No. 0 225 320.

[bb5] Sheldrick, G. M. (2008). *Acta Cryst.* A**64**, 112–122. 10.1107/S0108767307043930 18156677

